# Memory for diverse faces in a racially attentive context

**DOI:** 10.1186/s41235-021-00340-y

**Published:** 2021-11-04

**Authors:** Benjamin Uel Marsh, Deborah Revenaugh, Taylor Weeks, Hyun Seo Lee

**Affiliations:** 1 University of Tampa, 401 W. Kennedy Blvd., Tampa, FL 33606 USA; 2 Azusa Pacific University, Azusa, CA USA

**Keywords:** Cross-race effect, Other-race effect, Racial ambiguity, Racial categorization, Racism

## Abstract

Two experiments assessed how racial ambiguity and racial salience moderates the cross-race effect (CRE). In experiment 1, White and Black participants studied and identified the race of Asian, Black, Latino, and White faces that varied in ethnic typicality (high or low ET). For White participants, the CRE was larger when comparing high-ET White faces to high-ET other-race faces than low-ET other-race faces. Black participants showed a similar CRE reduction by ethnic typicality, but also showed a less prevalent CRE than White participants. Experiment 2 replicated experiment 1 procedures, but without the race identification task and only with White participants. Experiment 2 findings were comparable to experiment 1. Furthermore, experiment 2 showed a noticeably smaller CRE on Black faces than experiment 1, eliciting questions about increased racial salience amplifying the CRE. Results’ general implications and the conceptual roots that indirectly link the CRE and racism will be discussed.

## Significance statement

There have been seasons where our ability to see the equal humanity in those of another race has become top of mind of the American consciousness. Frustrations are voiced and complicated solutions are debated. Inadvertently, research on how we process the faces in other races may provide insights into countering racial divisions. We typically remember faces within our racial group better than faces outside of our racial group. This phenomenon known as the cross-race effect (CRE) is explained by individual differences in interracial contact as well as a myriad of socio-cognitive factors that likely affect the degree of attention one pays to a face’s racial category. In two experiments, this study manipulates attention to race in two ways: by presenting participants a diverse array of faces that vary in racial ambiguity and by having participants identify the race of each face studied. In general, memory differences were noticeably smaller when comparing racially unambiguous same-race faces to racially ambiguous other-race faces than racially unambiguous other-race faces. Furthermore, for White participants, identifying the race of each face was associated with a larger CRE for Black faces only. More interracial contact was associated with a smaller CRE in most cases. Perhaps, the CRE and racial divisions are perpetuated by common factors; namely segregative circumstances that limit quality interracial contact, and our tendency to sort entities into categories that reflect group cohesion over individuating qualities. Countering these factors may be akin to resolving how we see each other.

## Introduction

It is not uncommon for sociocultural differences, or worse, racial conflict to perpetuate a degree of cultural distance (Demes & Geeraert, 2014; Triandis, 2000) that undoubtedly leaves a cognitive mark. For instance, psychological scientists have consistently found that people are more accurate at recognizing same-race faces than other-race faces, a phenomenon known as the cross-race effect (CRE) (or the other-race effect and own-race bias). Typically, this memory difference is explained in terms of processing variations, wherein individuating facial features important for distinguishing one face from another—particular within racial/ethnic groups—are more likely to be encoded for same-race faces than other-race faces. Models meant to explain the CRE differ in the causal weight they assign to perceptual expertise in processing same and other-race faces, the process of categorizing faces into a shared social group, and the degree of motivation one has to encode individuating facial features (see the dual-route approach by Wan et al., 2015, the categorization-individuation model by Hugenberg et al., 2010, and the ingroup/outgroup model by Sporer, 2001). As evidence of socio-cognitive moderators of the CRE grow, theories attempt to accommodate how the effect varies not only by perceptual expertise (Hancock & Rhodes, 2008; Michel, et al., 2006; Tanaka, et al., 2013), but also by moderating the racial salience internally via cultural priming techniques (Marsh, 2021, Marsh et al., 2016, and Pauker et al., 2013) and externally through the presentation of racially ambiguous faces (Pauker et al., 2009; Maclin & Malpass, 2001). As a result, variations in racial salience either through contextual factors that highlight race, or racial ambiguity that blurs race remains a factor of interest.

Early research on the CRE attempted to associate racial attitudes with the effect, but support for a direct unmediated link was not found (see Meissner & Brigham, 2001 for a review). However, while racial attitudes may not relate directly to the CRE, an indirect relationship may exist through shared conceptual roots. Recently, Roberts and Rizzo (2021) outlined seven contributing factors to American racism. Two of those factors, categories and segregation, likely affect how much perceptual expertise someone has in other-race faces as well as their general conceptualization of racial categories. Hence, while research suggests that the CRE is not directly related to racism, it can highlight the cultural distance which is, in part, due to racist systems within American society, (specifically segregation).

Individuals typically have more exposure to same-race faces than other-race faces. Consequently, we learn what facial features are important for distinguishing one same-race face from another. In contrast, facial regions important for distinguishing one other-race face from another are poorly learned, decreasing the likelihood that encoded other-race faces will be distinguishable from others later. Despite the growth of racial diversity in America, the CRE is still consistently found among American participants. The persistence of racial segregation (Lichter et al., 2016) be it historically or economically driven (Rothstein, 2013, 2015)—or self-imposed through homophily (Fischer, 2008)—limits opportunities for interracial contact (Roberts & Rizzo, 2021) that could improve perceptual expertise of other-race faces and reduce prejudice attitudes (McKeown & Dixon, 2017; Paluck et al., 2019). However, some CRE research suggest that interracial contact has limited or no effect at all on recognition accuracy of other-race faces (see Wong et al., 2020). But, variations in the predictive relationship between interracial contact and face memory may be due variations in measuring methods (McKone et al., 2019; Singh et al., 2021) or even differences in what “quality” contact means in different interracial scenarios (i.e., White to Black, Asian to White, etc.). Nevertheless, a lack of heterogeneity in one’s racial experiences could feed into perceptual and socio-cognitive factors proposed to influence the CRE. Minimal contact with other races not only yields low perceptual expertise, but also could increase the likelihood of other-race individuals being recognized more for their group membership than their individuality.

As children, we begin to learn social categories as well as the supposed properties of those categories that potentially become essential markers of group membership (Rhodes et al., 2010; Roberts & Rizzo, 2021). This development leads to overgeneralization of learned group properties and has been linked to forms of stereotyping and prejudice (Bastian & Haslam, 2006; Pauker et al., 2016; Roberts & Rizzo 2021). In addition, our propensity to racially categorize faces is arguably an essential component of the CRE, as demonstrated by experiments utilizing racially ambiguous faces. The varied racial perception of racially ambiguous faces has mitigated the CRE in Black and White participants studying Black/White ambiguous faces (Pauker et al., 2009) and Latino participants studying Black/Latino ambiguous faces (Maclin & Malpass, 2001). Pauker et al. (2009) found that participants had poorer memory for racially ambiguous faces—created through a face morphing program—than for racially unambiguous same-race faces. More interestingly, they found that racially ambiguous faces labeled as same-race were better remembered than those labeled as other-race. When viewing a racially ambiguous face, the process may involve not only denoting a face as same-race or other-race, but also an attempt to identify the racial category of the face. To accomplish this task, individuals may turn to facial markers for racial/ethnic clues. Maclin and Malpass (2001) demonstrated this process by constructing computerized blends of Black and Latino faces. When hair characteristics were added to the face that could act as a marker for that particular race, the face was more likely to be perceived as the racial category congruent with the racial marker. If individuals scan other-race faces that are racially ambiguous for facial markers to aid in racial categorization, the behavior may promote the encoding of more individuating features compared to other-race faces that are easily categorized into a racial group. In fact, Marsh (2021) found that Asian participants showed the CRE among racially ambiguous faces (not computerized morphs, but naturally occurring faces), that is, they recognized racially ambiguous Asian faces better than racially ambiguous Latino and White faces, but showed no CRE among racially unambiguous faces. Perhaps, these participants within a racially diverse context are less likely to show the CRE, unless confronted with a scenario where a face’s racial category is in question and there is an impulse to determine which faces are Asian. Lucas and et al., (2011) found that electrophysiological activity associated with processing individuating facial features (i.e., N200 and P2 amplitudes) was greater for racially atypical Black faces (other-race) than stereotypical Black faces. Moreover, atypical faces were better remembered than the stereotypical faces. Note that Rhodes et al. (2010) is an exception to this trend; however, they used Asian/White ambiguous faces studied by White participants, a racial context that may not be susceptible to some socio-cognitive manipulations as suggested by Wan et al., (2015).

While past studies suggest that the type of other-race face impacts the moderation of the CRE (Marsh, 2021; Wan et al., 2015), they also suggest that factors impacting racial salience may have a moderating role in the CRE. Thus, this study assesses how the size of the CRE varies by the type of other-race face and by the racial ambiguity of those other-race faces. Racially unambiguous same-race faces will be compared to racially ambiguous other-race faces. If the size of the CRE is smaller for other-race faces that are racially ambiguous compared to racially unambiguous, then racially ambiguous other-race faces may be receiving more individuating processing than racially unambiguous other-race faces. Moreover, these effects are tested within two different experimental contexts. One context (experiment 1)—that consists of data from a larger project, some of which was published in Marsh (2021)—explicitly draws attention to race by having participants identify the race of each face they study. In the other context (experiment 2), the racial identification task was not used. Differences between the experiments in the size of the CRE are also explored.

## Experiment 1

### Methods

#### Participants and design

This study was a 2 (Ethnic Typicality: High and Low) × 4 (Race of Face: Asian, Black, Latino, and White) within-subjects design. Fifty-six White (Female = 43; Male = 12; Prefer Not to Answer = 1) and 29 Black (Female = 20; Male = 8; Prefer Not to Answer = 1) college students at a private university in Los Angeles County, California, participated in this experiment for participation credit in a lower division psychology course. White and Black participants mean age was 19.38 (SD = 3.71) and 21.74 (SD = 5.71) years old respectively.

#### Procedure and materials

The experiment was administered via a desktop computer with a 22″ screen using iMotions biometric platform equipped with a Tobii X2-60 eye-tracker. One-hundred and twenty-eight faces that equally varied by race (i.e., Asian, Black, Latino, and White) were chosen from the Chicago face database (Ma et al., 2015). Half of the faces were high in ethnic typicality and the other half were low in ethnic typicality according to the database’s ratings (Ma et al., 2015). Faces were similar in attractiveness, unusualness, and age (see Marsh 2021). Two random orders of faces were used in the experiment. Participants studied 64 faces half of which were male (and half female) and half of which were high (and half low) in ethnic typicality. Each race was represented with 16 faces, meaning that eight faces (4 male and 4 female) were Asian and high in ethnic typicality, while another eight faces were Asian and low in ethnic typicality. Participants were told to study each face carefully because their memory for the faces would be tested later. Faces were presented in color one at a time for 6 s. After each face, participants were prompted to identify the face’s race choosing one of five options: Asian, Black, Latino, White, or Other (see Table 1 for subjects’ responses). After studying half of the faces (32 faces), subjects were prompted to either type five words that described their American identity or five words that described their Ethnic identity. The effects of cultural priming on face recognition were not assessed in these analyses; thus, analyses were conducted controlling for the between-subjects variable, priming condition. After studying the faces, participants received the test phase wherein all 64 study faces were intermixed with 64 new faces. Test faces were displayed individually and on a grayscale rather than in color. They remained on the screen until the participant indicated whether they had seen the face during the study phase and rated their confidence (1 = Not at all confident; 5 = Very confident) in their answer. After the test phase, participants took a questionnaire covering basic demographics as well as measuring their exposure to individuals from each of the four relevant racial groups using an adapted version of Hancock and Rhodes (2008) racial contact questionnaire (see Table 2 for exposure ratings).

**Table 1:** Participants’ mean (*standard devation*) proportion of faces racially categorized accurately by racial/ethnic group and ethnic typicality

Race of face	Ethnic typicality	Participants	
		White	Black
Asian	High	.982 *(.060)*	.965 *(.065)*
	Low	.363 *(.235)*	.478 *(.189)*
Black	High	.997 *(.016)*	.995 *(.023)*
	Low	.544 *(.205)*	.525 *(.201)*
Latino	High	.805 *(.190)*	.715 *(.185)*
	Low	.187 *(.163)*	.245 *(.217)*
White	High	1.00 *(.000)*	.995 *(.023)*
	Low	.464 *(.248)*	.418 *(.257)*

**Table 2:** Participants’ mean (*standard devation*) racial exposure ratings for Asian, Black, Latino, and White individuals by Experiment

Racial Group	Experiment 1		Experiment 2
	Participants		
	Black	White	White
Asian	2.98 *(1.29)*	4.00 *(.954)*	3.97 *(1.26)*
Black	4.88 *(1.20)*	3.96 *(.957)*	3.83 *(1.14)*
Latino	4.10 *(1.22)*	4.55 *(.961)*	4.42 *(1.12)*
White	4.69 *(.795)*	5.30 *(.561)*	5.41 *(.659)*

## Results and discussion

Differences in exposure to individuals of the same-race and another race was expected to moderate the CRE. Thus, it was important to first determine the extent to which exposure to same-race people was greater than that of the other-race people. A repeated measures ANOVA showed a main effect of exposure in White, *F*(3, 162) = 32.60, *p* < 0.001, ${\eta }_{p}^{2}$= 0.376, and Black Participants, *F*(3, 78) = 16.73, *p* < 0.001, ${\eta }_{p}^{2}$= 0.392. Contrasts showed that White participants had significantly more exposure with White individuals than Asian, *F*(1, 54) = 69.37, *p* < 0.001, ${\eta }_{p}^{2}$= 0.562, Black, *F*(1, 54) = 61.70, *p* < 0.001, ${\eta }_{p}^{2}$= 0.533, and Latino individuals, *F*(1, 54) = 22.07, *p* < 0.001, ${\eta }_{p}^{2}$= 0.290. Additional comparisons showed that White participants reported more exposure with Latino individuals than Asian, *t*(55) = 3.39, *p* = 0.001, and Black individuals, *t*(55) = 4.08, *p* = 0.001. As for Black participants, contrasts showed more exposure with Black individuals than Asian, *F*(1, 26) = 47.88, *p* < 0.001, ${\eta }_{p}^{2}$= 0.648, and Latino individuals, *F*(1, 26) = 5.92, *p* = 0.022, ${\eta }_{p}^{2}$= 0.185. Additional comparisons showed that Black participants reported less exposure to Asian individuals than Latino, *F*(1, 26) = 15.12, *p* = 0.001, ${\eta }_{p}^{2}$= 0.368, and White individuals, *F*(1, 26) = 38.88, *p* < 0.001, ${\eta }_{p}^{2}$= 0.599.

**Fig. 1:**
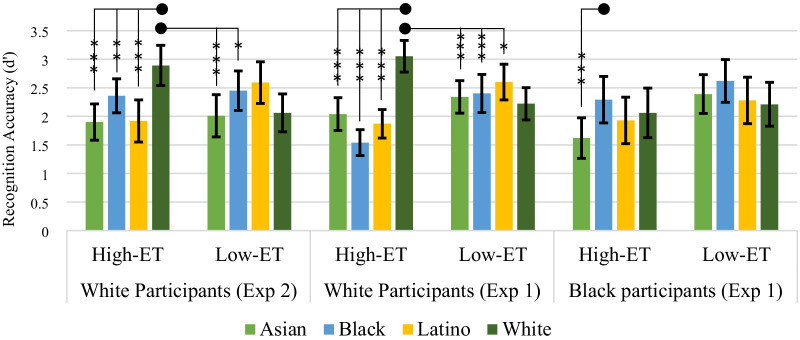
Mean recognition accuracy for each face type with 95% confidence intervals, by participant and experiment. *Note*: *p > .0167 & < .05, **p < .01, ***p < .001

Separate repeated measures ANOVAs were conducted on recognition accuracy (*d*′) comparing high-ET same-race faces to high-ET and low-ET other-race faces for White and Black participants (see Fig. 1 for means). Furthermore, supplemental analyses were conducted entering differences in exposure to same-race and the relevant other-race persons as a covariate and are presented in brackets. Planned contrasts were assessed with a Bonferroni correction alpha level of 0.0167, representing the three relevant comparisons necessary to test for the CRE for each other-race face.

### High-ET same-race and high-ET other-race

In White participants, comparing high-ET White faces to high-ET other-race faces, the Hyunh–Feldt (Epsilon = 0.931, *p* = 0.012) analysis, used to correct a violation of sphericity, found a race of face main effect, *F*(2.79, 150.86) = 27.44, *p* < 0.001, ${\eta }_{p}^{2}$= 0.337. Planned contrasts showed that participants recognized high-ET White faces better than high-ET Asian, *F*(1, 54) = 35.16, *p* < 0.001, ${\eta }_{p}^{2}$= 0.394 [*F*(1, 53) = 14.41, *p* < 0.001, ${\eta }_{p}^{2}$= 0.214], Black, *F*(1, 54) = 72.60, *p* < 0.001, ${\eta }_{p}^{2}$= 0.573 [*F*(1, 53) = 34.86, *p* < 0.001, ${\eta }_{p}^{2}$= 0.397], and Latino faces, *F*(1, 54) = 41.27, *p* < 0.001, ${\eta }_{p}^{2}$= 0.436 [*F*(1, 53) = 43.80, *p* < 0.001, ${\eta }_{p}^{2}$= 0.452]. These results suggest that the CRE occurred consistently for each other-race face. Also, it is noteworthy that the effect size decreased in a way that suggests exposure moderated the effect except in the case of Latino faces.

Another interest was whether the size of the CRE varied significantly by the type of other-race face. Thus, a repeated measures ANOVA was conducted on the difference scores between high-ET White and high-ET other-race faces. The Huynh–Feldt analysis (Epsilon = 0.870, *p* = 0.002), used to correct a violation of sphericity, found a main effect of CRE comparison (i.e., White Asian, White Black, and White Latino), *F*(1.74, 93.97) = 4.19, *p* = 0.023, ${\eta }_{p}^{2}$= 0.072. Planned contrasts showed that the CRE was significantly larger for Black faces than Asian, *F*(1, 54) = 10.32, *p* = 0.002, ${\eta }_{p}^{2}$= 0.161, and Latino faces, *F*(1, 54) = 4.59, *p* = 0.037, ${\eta }_{p}^{2}$= 0.078, but this last difference was not a significant at the Bonferroni correction level (i.e., *α* = 0.0167). This suggests that discriminability of Black faces appears to be more difficult for these White participants which could be a testament to the quality of their perceptual expertise on those faces. However, exposure ratings were similar for Black and Asian faces, suggesting that these effects are either due to aspects of exposure not reflected in the scale or socio-cognitive mechanism that hinder processing of Black faces more so than Asian faces.

For Black participants, the CRE was not as prevalent as White participants. Comparing high-ET Black faces to high-ET other-race faces, analysis found a race of face main effect, *F*(3, 81) = 3.57, *p* = 0.017, ${\eta }_{p}^{2}$= 0.117. Planned contrast showed that participants recognized Black faces better than Asian faces, *F*(1, 27) = 20.28, *p* < 0.001, ${\eta }_{p}^{2}$= 0.429 [*F*(1, 24) = 5.35, *p* = 0.030, ${\eta }_{p}^{2}$= 0.182], but not Latino, *p* = 0.205, or White faces, *p* = 0.449. Note that controlling for exposure to Asian faces not only reduced the size of the CRE, but also changed the difference to nonsignificant at the Bonferroni correction level (i.e., *α* = 0.0167). Furthermore, the low exposure to Asian individuals stands out among the other types of other-race faces for which no CRE was found. Moreover, this result is a partial replication of Gross (2009) who found that Black participants exhibited the CRE for Asian and Latino faces, but not White faces in a diverse face array.

### High-ET same-race and low-ET other-race

Comparing high-ET White faces to low-ET other-race faces, there was also a race of face main effect, *F*(3, 162) = 6.52, *p* < 0.001, ${\eta }_{p}^{2}$= 0.108. Planned contrasts showed that participants recognized high-ET White faces better than low-ET Asian, *F*(1, 54) = 23.64, *p* < 0.001, ${\eta }_{p}^{2}$= 0.305 [*F*(1, 53) = 16.59, *p* < 0.001, ${\eta }_{p}^{2}$= 0.238], Black, *F*(1, 54) = 11.64, *p* = 0.001, ${\eta }_{p}^{2}$= 0.177 [*p* = 0.079] and Latino faces, *F*(1, 54) = 5.93, *p* = 0.018, ${\eta }_{p}^{2}$= 0.099 [*F*(1, 53) = 7.79, *p* = 0.007, ${\eta }_{p}^{2}$= 0.128], but not at the Bonferroni correction level. Note that exposure reduced the size of the CRE for Asian faces, eliminated the CRE for Black faces, and exacerbated it for Latino faces. The exposure effect with Latino faces was shown from a different statistical vantage point (i.e., a regression analysis) in Marsh (2021). In either case, it suggests that an increase in exposure may improve one’s ability to racially identify these racially ambiguous Latino faces in a way that hinders the processing of their individuating features. When assessing the size of the CRE by type of other-race face, there was no main effect of CRE comparison, *p* = 0.324. This suggests that the size of the CRE did not vary by race type in racially ambiguous other-race faces.

In addition, a 2 (Ethnic Typicality: High and Low) X 3 (CRE Comparison) repeated measures ANOVA was conducted to assess the effect that racial ambiguity has on the size of the CRE while ignoring type of other-race face. The analysis found a main effect of ethnic typicality, *F*(1, 54) = 35.31, *p* < 0.001, ${\eta }_{p}^{2}$= 0.395, wherein the CRE was larger when comparing high-ET White faces to high-ET other-race faces (*M* = 1.24, SE = 0.146) than low-ET other-race faces (*M* = 0.619, SE = 0.144). A similar analysis was not necessary for Black participants, because when comparing high-ET Black faces to low-ET other-race faces, there was no race of face main effect, *p* = 0.724, thus no CRE. These findings suggest that racial ambiguity affords other-race faces enough individuating processing to mitigate the CRE in White participants and eliminate it in Black participants. One plausible explanation of these effects is that racial clarity about an other-race face facilitates access to the face’s social category and its deindividuation. In contrast, racial ambiguity limits access to the face’s social category, therein increasing the potential for the face to be individuated. Also, exposure did not moderate the CRE for high-ET and low-ET Latino faces in White participants, even though participants had the most exposure with Latino individuals than Asian or Black individuals. This race-specific discrepancy suggests either exposure’s mitigating effect could be moderated by the type of other-race and the experimental context, or that exposure ratings do not represent the same quality of contact for each race.

However, these effects occurred under circumstances that highlighted the racial category of each face. In fact, the racial identification task was expected to exacerbate the CRE at least among high-ET faces. Thus, there is a question of whether the results will replicate when presenting the same procedure without the racial identification task. Moreover, would the effect size of the CRE comparisons be noticeably smaller once the racial identification task is removed?

## Experiment 2

In experiment 2, the race identification task was removed to assess whether the effects found in White participants during experiment 1 will replicate under conditions that do not explicitly direct attention to the race of each face. In addition, treating experiment type as a subject variable, we will compare the size of the CRE under both experimental conditions.

### Methods

#### Participants and design

The design was identical to experiment 1. Forty-four (Female = 36; Male = 2; Prefer Not to Answer = 6) White American college students from a private university in Los Angeles County, California, participated in this experiment for participation credit in a lower division psychology course. The mean age was 18.53 years (SD = 0.830).

#### Materials and procedures

All materials and procedures were the same as experiment 1 with one exception. Participants did not have the race identification task. Thus, study faces were presented without any intermediate task.

### Results and discussion

Again, exposure is expected to moderate the CRE, thus it was necessary to assess whether participants reported more exposure with same-race individuals than other-race individuals. A repeated measures ANOVA showed a main effect of exposure, *F*(3, 114) = 20.68, *p* < 0.001, ${\eta }_{p}^{2}$= 0.352, and contrasts showed that White participants had significantly more exposure with White individuals than Asian, *F*(1, 38) = 31.06, *p* < 0.001, ${\eta }_{p}^{2}$= 0.450, Black, *F*(1, 38) = 48.82, *p* < 0.001, ${\eta }_{p}^{2}$= 0.562, and Latino individuals, *F*(1, 38) = 15.63, *p* < 0.001, ${\eta }_{p}^{2}$= 0.292. Additional comparisons showed that participants reported more exposure with Latino individuals than Asian, *t*(39) = 3.39, *p* = 0.018, and Black individuals, *t*(39) = 3.42, *p* = 0.001.

#### High-ET same-race and high-ET other-race

A repeated measures ANOVA on recognition accuracy (*d*’) found a race of face (White, Asian, Black, and Latino) by ET (high and low) interaction, *F*(3, 132) = 8.88, *p* < 0.001, ${\eta }_{p}^{2}$= 0.168 (see Fig. 1 for means). Again, supplemental analyses controlling for differences in exposure are presented in brackets, and planned contrasts are assessed at a Bonferroni correction alpha level of 0.0167. Comparing high-ET White faces to high-ET other-race faces, a repeated measures ANOVA analysis, with recognition accuracy (*d*’) as the dependent variable, found a race of face main effect, *F*(3, 132) = 12.03, *p* < 0.001, ${\eta }_{p}^{2}$= 0.215. Planned contrasts showed that participants recognized high-ET White faces better than high-ET Asian, *F*(1, 44) = 33.02, *p* < 0.001, ${\eta }_{p}^{2}$= 0.429 [*F*(1, 37) = 11.26, *p* = 0.002, ${\eta }_{p}^{2}$= 0.233], Black, *F*(1, 44) = 7.46, *p* = 0.009, ${\eta }_{p}^{2}$= 0.145 [*p* = 0.091], and Latino faces, *F*(1, 44) = 28.47, *p* < 0.001, ${\eta }_{p}^{2}$= 0.393 [*F*(1, 37) = 14.43, *p* = 0.001, ${\eta }_{p}^{2}$= 0.281]. These results replicate experiment 1 when exposure is not controlled for. When exposure is included as a covariate there is a reduction in the CRE for Asian faces and an elimination of the CRE for Black faces. In contrast to experiment 1, exposure also mitigated the CRE for Latino faces.

More analyses were conducted to test whether the size of the CRE varied significantly by the type of other-race face. A repeated measures ANOVA was conducted on the difference scores between high-ET White and high-ET other-race faces. The analysis found a main effect of CRE comparison (i.e., White Asian, White Black, and White Latino), *F*(2, 88) = 3.96, *p* = 0.022, ${\eta }_{p}^{2}$= 0.083. Planned contrasts showed that the CRE was significantly smaller for Black faces than Asian faces, *F*(1, 44) = 7.00, *p* = 0.011, ${\eta }_{p}^{2}$= 0.137, and Latino faces, *F*(1, 44) = 5.67, *p* = 0.022, ${\eta }_{p}^{2}$= 0.114, but not at the Bonferroni correction level (i.e., *α* = 0.0167). While experiment 1 and 2 are largely similar (see Fig. 1), they contrast with regards to the CRE for Black faces. For White participants, in experiment 1, the CRE was the largest for Black faces but in experiment 2 it was the smallest.

#### High-ET same-race and low-ET other-race

Comparing high-ET White faces to low-ET other-race faces, there was a race of face main effect, *F*(3, 132) = 5.83, *p* = 0.001, ${\eta }_{p}^{2}$= 0.117. Planned contrasts showed that participants recognized high-ET White faces better than low-ET Asian, *F*(1, 44) = 14.59, *p* < 0.001, ${\eta }_{p}^{2}$= 0.249 [*F*(1, 37) = 11.88, *p* = 0.001, ${\eta }_{p}^{2}$= 0.243], and Black faces, *F*(1, 44) = 4.58, *p* = 0.038, ${\eta }_{p}^{2}$= 0.094 [*p* = 0.684], but not at the Bonferroni correction level (i.e., *α* = 0.0167). There was no difference between high-ET White faces and low-ET Latino faces, *p* = 0.100 [*p* = 0.298]. When assessing the size of the CRE with low-ET other-race faces, there was a main effect of CRE comparison, *F*(2, 88) = 3.71, *p* = 0.028, ${\eta }_{p}^{2}$= 0.078. Planned contrasts showed that the CRE was significantly larger for Asian faces than Latino faces, *F*(1, 44) = 9.194, *p* = 0.004, ${\eta }_{p}^{2}$= 0.173; an unsurprising effect considering there was no CRE for Latino faces. In addition, the CRE did not differ in Asian and Black faces (*p* = 0.051). Note that these results represent a minimally less prevalent CRE in experiment 2 than experiment 1. However, it is unclear whether this is due to spurious variations between experiments or the lack of explicit attention to the race of the faces.

Additionally, a 2 (Ethnic Typicality: High and Low) X 3 (CRE Comparison) repeated measures ANOVA was conducted to assess the effect that racial ambiguity has on the size of the CRE while ignoring the type of other-race face. The analysis found a main effect of ethnic typicality, *F*(1, 44) = 6.90, *p* = 0.012, ${\eta }_{p}^{2}$= 0.136, wherein the CRE was larger when comparing high-ET White faces to high-ET other-race faces (*M* = 0.836, *SE* = 0.135) than low-ET other-race faces (*M* = 0.531, *SE* = 0.169). This finding replicates experiment 1 and suggests that low-ET other-race faces receive more individuating processing than high-ET other-race faces.

#### Size of CRE by race identification task

**Fig. 2:**
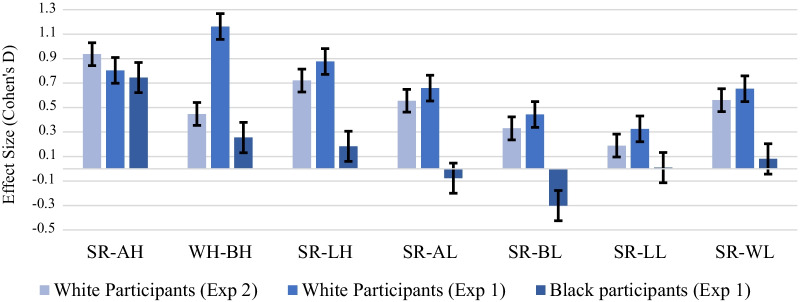
Effect size by CRE comparison, participant and experiment. Error bars are standard errors

Lastly, a repeated measures ANOVA was conducted on the recognition accuracy (*d*′) of high-ET faces with experiment type as a factor. The Huynh–Feldt analysis (Epsilon = 0.986, *p* = 0.045), used to correct a violation of sphericity, found a race of face by experiment interaction, *F*(2.95, 289.95) = 6.73, *p* < 0.001, ${\eta }_{p}^{2}$= 0.064. Planned contrasts showed that experiments differed in the size of the CRE for Black faces, *F*(1, 98) = 15.94, *p* < 0.001, ${\eta }_{p}^{2}$= 0.140 (see Fig. 2 for CRE effect sizes by experiment). Experiment 1, that used the race identification task, had a larger CRE between high-ET White and Black faces than experiment 2, which did not have the race identification task. It is important to note that while this finding suggests an association between explicitly directing attention to the race of face and an augmented CRE for one type of other-race face, these groups were not created via random assignment, thus limits our ability to make causal claims about the effect. Hence, it stands as an observation that requires further testing. There was no significant differences between experiments when comparing high-ET White faces to low-ET faces, *p* = 0.570.

## General discussion

These two experiments provide a few insights into perceptual and socio-cognitive factors involved in the CRE. While White participants demonstrated a consistent CRE for all racially unambiguous other-race types in both experiments, Black participants showed a more limited CRE similar to Black participants in Gross (2009) and Asian and Latino participants in other studies (Gross, 2009; Marsh, 2021). The difference in the prevalence of the CRE between White participants and non-White participants in America is more clearly revealed within a racially diverse experimental context. In Marsh (2021), the differences may be better explained by socio-cognitive differences between White, Asian, and Latino participants. However, in this study, Black participants’ limited CRE appears to be more exposure based given the relatively low exposure rate for same-race individuals and that the CRE was only shown for faces with a considerably low exposure rate (i.e., Asian faces). However, it is plausible that the small sample of size limited the CRE prevalence in Black participants. At the least, the result here and elsewhere (Gross, 2009; Marsh, 2021; Wan et al., 2015) suggests there are race or culture specific moderators of the CRE that have yet to be unpacked.

While differences in same-race and other-race exposure mitigated the CRE, other-race faces with similar exposure ratings (i.e., Asian and Black faces in experiment 1) yielded noticeably different sizes of the CRE; a subtle note to either the involvement of other factors or race-specific discrepancies in what it means to have a quality interaction with the two racial groups. Not to mention, in one case, exposure appeared to exacerbate the CRE in White participants for racially ambiguous Latino faces, suggesting that increased interracial contact may have a more complicated effect on the CRE in racially diverse cultural contexts. Perhaps, there is a quality of interracial contact that merely improves our ability to racially identify and categorize rather than individuate.

Racially ambiguous faces reduced the CRE in White participants and eliminated it in Black participants. The variation in this effect is likely due to three intermingling factors. Some racially ambiguous faces are more likely to be identified as a same-race face, a typical occurrence for the low-ET Latino faces used (see Marsh, 2021). In addition, racial ambiguity may have afforded better processing due to the perceiver's attempt to identify features that could reveal the racial identity of the face (Maclin & Malpass, 2001). However, variations in the effect could be simpler in that naturally occurring racial ambiguity may afford a degree of distinctiveness that racially unambiguous faces do not have (Lucas et al., 2011).

There were minimal differences between experiments, but one key deviation. In White participants, when attention was explicitly drawn to race, the CRE was noticeably larger for Black faces, even with similar exposure ratings for Asian and Black individuals,. However, when the racial identification task was removed, the CRE was smallest for Black faces despite the similar exposure ratings between White participants in experiment 1 and 2. This contrast between the experiments is perhaps spurious. However, this singular race-specific effect should elicit inquisition into how different types of other-race faces are appraised among individuals with varying degrees (quantity and quality) of interracial contact and within experimental contexts where racial salience is manipulated along with the dimming of the Other’s individual qualities.

## CRE insights into racism

While the CRE may not be about negative racial attitudes hindering individuating processing of other-race faces (Meissner & Brigham, 2001), it is related to divisive practices within our society, particularly segregation and “diversity” without positive engagement. Due to these factors, there is a persistent degree of cultural distance between racial groups that limits our perceptual expertise of other-races and facilitates our inability to notice the individuality of other-race persons. Of course, all interracial contact is not equal in quality or affective valence, thus some contact will be more productive than others (McKeown & Dixon, 2017). Nonetheless, little contact neither aids our other-race perceptual abilities nor our racial reconciliation efforts. The CRE is partially about the hyper salience of race in our society that makes it difficult to divert attention away from it, without the aid of contextual entities that either blur racial lines (i.e., racial ambiguity) or redirect our attention to a prominent social category shared with the other-race individual (Marsh, 2021; Marsh et al., 2016; Pauker et al., 2013). Our tendency to divide others and ourselves into social cohorts promulgates division seen not only in how we deal with the other-race person, but also in how well we remember their face.

The CRE demonstrates the depth to which cultural distance mediates our experience of others. However, it also suggests that racialized disparities in perception can be overcome. If we increase the quantity, but more importantly, the quality of our interracial contact, we can improve our ability to see others’ individuating features not only outshine their racial category, but also correct our prejudices.

## Data Availability

Faces used has been stored with Open Science Framework at 10.17605/OSF.IO/ZAKYT. Due to privacy statements in the consent form, the data set is not publicly available.
